# The Review on the Role of Ambiguity of Tolerance and Resilience on Students’ Engagement

**DOI:** 10.3389/fpsyg.2021.828894

**Published:** 2022-01-13

**Authors:** Miao Yu, Hongliang Wang, Guoping Xia

**Affiliations:** ^1^College of Teacher Education, Baoding University, Baoding, China; ^2^College of Artificial Intelligence, Baoding University, Baoding, China; ^3^School of Humanities and Social Sciences, Beijing Institute of Technology, Beijing, China

**Keywords:** ambiguity of tolerance, resilience, students’ engagement, positive psychology, individual differences

## Abstract

Due to the arrival of positive psychology (PP) in the development of teaching, the construct of engagement has been thrived and got a notable function in the educational arena. Alternatively, numerous individual differences, containing ambiguity of tolerance, have been taken into consideration as a result of the key role they can play in the process of learning, and thus, on different facets of the learners’ engagement. Furthermore, resilience is recommended to be an alternate and effective way of engaging English as a foreign language (EFL) learners. Also, it is a significant feature of the human adaptation system in which students can efficaciously manage and tackle stressful involvements despite their troubles and disasters. Given the eminence of both ambiguity tolerance and resilience in educational settings and the fact that little attention has been given to these constructs in foreign language learning, the present review makes an effort to scrutinize the impact of ambiguity of tolerance and resilience on EFL learners’ engagement. Succinctly, the fundamental roles of ambiguity tolerance and resilience in learners’ engagement were confirmed, and consistent with the conclusions drawn from the present review, some suggestions are set forth concerning the implications of this paper.

## Introduction

A significant number of weak students have personal and contextual reasons for dropping out of school, and dropout signifies disengagement from school and is a rapidly growing phenomenon (Lippman and Rivers, 2008). Therefore, learners’ engagement is one of the most important problems in scholastic practices and it alludes to learners’ motivation and active participation in scholastic organization-centric programs (Mercer, 2019; Wang et al., 2021). It is demonstrated in the consolidation of inspiration and practical actions to achieve anticipated educational results (Pagán, 2018). Within the previous decades, engagement, with its numerous transformers; for instance, school, learner, or classroom is an exhortation for an issue that has turned into very widespread aspects in learning systems (Fredricks and McColskey, 2012). Higher education organizations can motivate students to utilize their skills and educational chances and institutions that allow them to be engaged in an active manner (Xie and Derakhshan, 2021) as it is characterized by the integration of enthusiasm and practical movements to influence the estimated learning consequences (Fredricks and McColskey, 2012). It is mentioned that students are more likely to attain greater degrees of learning when they take part in educational advancement in an active manner and are interested in their scholastic learning (MacIntyre and Mercer, 2014; Wang et al., 2021). Moreover, actively taking part in assignments and activities is known as engagement, and it provides a system for comprehending learning issues like dropping out of school (McKelvey and Zaring, 2018) and an important element in improving scholastic performance is scholastic engagement in learning activities (Thomas and Allen, 2021). Scholastic engagement is the entirety of what makes up a learner, with practices that uniquely fulfill a learners’ identity. This is constituted of three elements related to behavior, emotions, and cognition, all of which are logically interconnected to achieve overall school achievement (Adeniji et al., 2020). In the education and learning of new languages, the emotional aspect has an important part and can influence two other aspects that are directly connected to the psychology of language education (Pishghadam et al., 2021). Scholastic engagement is especially significant, and so, determining its successful elements can add to high academic success. There has been new attention to studying elements associated with learners’ engagement (Blum and Libbey, 2004; Klem and Connell, 2004) as it is becoming more and more recognized as an important factor in engaging learners for high degrees of scholastic achievement, and to keep away from deconstructive results like dropouts (Shochet et al., 2006; Bond et al., 2007).

Since numerous educators not only in different fields of study, but also in language education have acknowledged the trials of keeping learners engaged and concentrated on the ways to deal with in case of any challenges and demanding situations (Mercer and Dörnyei, 2020). It has been stated that those learners who are more involved in their classroom are likely to have theoretical information, employ practical learning approaches, accomplish high educational results, begin sensible social interactions, and enjoy pleasing motivational prominence in the classroom, so the improvement of consistent and applied approaches and tactics for learners’ engagement in class has come to be significance for scholars (Zepeda et al., 2020). Subsequently, a new line of psychology, labeled positive psychology (PP) which stands for postmodern psychology tries to focus on the way to increase the learners’ engagement (Zakeri et al., 2010). While conventional psychology emphasized uncommonness, sickness, and pathology, PP has to do with the scientific research on individuals’ strengths and competencies, like wellbeing, aspiration, resilience, and joy (Nolan et al., 2014). An early field in PP, resilience is considered as a multifaceted concept including self-confidence, hardiness, and supportive resources (Windle et al., 2011). Moreover, it is a significant element that influences learners’ tolerance of ambiguity and enhances scholastic engagement (Avarandeh et al., 2020). Scholastic resilience alludes to learners’ capability of overcoming issues, impediments, and challenges of day-to-day scholastic activities, like poor grades, exam pressure, homework difficulties, and negative feedback in educator-learner connections, competition, and loss of inspiration (Yu et al., 2019). Furthermore, scholastic resilience is aimed at constructive life processes and the mental empowerment of learners. It is characterized as the capability of effectively adjusting constructively to difficult or challenging situations (Zakeri et al., 2010). Effective resilience interference programs have the potential to enhance learners’ aspirations, psychological health, and course performance (Shellman and Hill, 2017) by assisting learners to control and decrease stress and depression symptoms (Shatkin et al., 2016; Houston et al., 2017). Resilience allows students to overcome educational difficulties in the long term without giving up easily. At some point, all individuals will encounter anxiety, risks, and misfortunes to different extents. These difficult times can adversely affect a person’s well-being and growth. According to the literature, resilience decides the difference between those who keep on effectively and those who do not (Zakeri et al., 2010). Simmons et al. (2018) declared that even though many authors have characterized resilience differently, it is generally based on two main notions, namely, hardship and constructive adjustments. Resilience can thus be regarded as the capability of constructively adjusting when hardships are encountered.

The English learning process can be a bit ambiguous since it deals with uncertain linguistic and cultural patterns that can confuse new language learners and EFL students typically encounter ambiguous circumstances while learning a foreign language. Tolerance for ambiguity is associated with learning a second or foreign language (Li and He, 2016). In the EFL context, learners are inclined to have problems in comprehending new construction and sense planned in a foreign language, and exposure to various unknown, intricate, and ambiguous approaches can create confusion. In language education, the capability of managing new uncertain circumstances without feeling frustration is known as ambiguity tolerance (Chu et al., 2015). In such circumstances, the person’s personality decides the degree to which this unpredictable circumstance can be effectively addressed (Hancock and Mattick, 2020). In this intricate cycle, achievement has numerous distinct factors, such as tolerance for ambiguity when learning a new language. Ambiguity tolerance in learning English as a foreign language (EFL) is a contribution to the success of the learning since it has an important effect on students’ language acquisition abilities (Brown, 2000).

Tolerance of ambiguity is one of those personality factors represented in distinct forms in modern psychology literature, along with other related pedagogical factors that educators can use to learn more about the significance of students’ characteristics (Li and He, 2016). As a personality attribute, tolerance of ambiguity involves how an individual deals with debatable circumstances and unclear and inaccurate clues (Chu et al., 2015). Embracing unpredictability as an aspect of life, the capability of surviving without complete information, and the inclination toward beginning a direct action without obvious outcomes all fall under tolerance for ambiguity (Iannello et al., 2017). It alludes to the cycle by which an individual deals with information in an unpredictable circumstance and reacts to that information in a series of intellectual, emotional, and behavioral responses (Shaterian Mohammadi et al., 2014).

It should be noted that ambiguity tolerance is considered as one of the main factors that motivate language students to actively take part in classroom activities; therefore, the higher the level of ambiguity tolerance, the higher the level of engagement (Zarfsaz and Takkac, 2014). People with a low tolerance for ambiguity are inclined to discern unpredictability and ambiguous circumstances as threats that result in anxiety, delay, negation, suppression, and avoidance (Furnham and Marks, 2013). In addition, learners who are less tolerant of ambiguity demonstrate less affinity to difficulty, which means that they are inclined to avoid hard scholastic assignments or scholastic challenges and regard them as challenging. Therefore, a learners’ reception of scholastic challenge is directly proportional to ambiguity tolerance (Bardi et al., 2009; Wang and Guan, 2020). Learners with high ambiguity tolerance are more comfortable when encountering uncertain cases and unpredictability during various educational circumstances. Thus, tolerance for ambiguity can affect learner performance (Alahdadi and Ghanizadeh, 2017). People who tolerate ambiguity tend to be productive and enjoy dealing with intricate, innovative, and unpredictable circumstances (Chiang, 2016). According to MacIntyre and Mercer (2014), many language teachers are conscious about the significance of enhancing personal students’ experiences of language education by assisting them with building and sustaining their inspiration, persistence, resiliency, and constructive feelings required for the long-run course of foreign language education. Additionally, many scholars have categorized ambiguity tolerance as a crucial and indispensable disposition and behavior (Dewaele and Li, 2013; Atamanova and Bogomaz, 2014). This personality can influence students in a variety of ways, including their language proficiency, educational techniques, and class participation. Learners who are less tolerant of ambiguity in language education require more support and motivation from their educators (Chu et al., 2015). Moreover, resilience has been lately scrutinized in general education (Martin and Marsh, 2006), although based on the researchers’ knowledge, little is conveyed about the function of resilience in the field of foreign language learning on the one hand and its role regarding language engagement on the other hand. Therefore, one of the foremost objectives of the present paper was to bring these two constructs, namely resilience, and ambiguity of tolerance into concentration in language education and research, and examine their main role on language learners’ engagement.

## Review of the Literature

### Learner Engagement

Students’ engagement is related to how long the students successfully engage in-class tasks and activities. Furthermore, it is defined by how much the student is engaged in learning in a traditional educational cycle. It refers to the time, effort, and energy exerted in the educational task (Chang et al., 2016). As stated by Thomas and Allen (2021), engagement in the context of learning is the endeavor, care, resources, and abilities utilized by learners to carry out assignments inside and outside the class, and the approaches and strategies teachers utilize to motivate students to be engaged in the instructional assignments. Learner engagement is considered a multifaceted construct, and it is categorized into different types: Behavioral (physical), emotional, cognitive, and Social (Rangvid, 2018). Behavioral engagement, for example, is regarded as students’ participation in learning activities, their engagement level, and active involvement in the instructional cycles (Hiver et al., 2021; Wang and Derakhshan, 2021). Emotional engagement is believed to have an important impact on various components of engagement. This is because the abstract mentality or differentiators that learners instill in their classes or through relevant tasks are fundamental to the various components of engagement (Henry and Thorsen, 2020). Therefore, emotional engagement is related to the learners’ viewpoints regarding the educational environment, the people in that specific environment, the tasks, and their collaboration in learning (Reeve, 2012). According to Maroco et al. (2016), cognitive (intellectual) engagement alludes to the learners’ motivation and dedication to understanding and mastering intricate notions and hard scholastic abilities. Emotional engagement alludes to a learner’s constructive and deconstructive responses to peers, school, school belonging, educator relationships, and beliefs regarding the value of school education. Social engagement is defined by taking into account the social challenges and contributions recognizable in the educational network, such as relationships with interrogators and the types of these social relationships (Mercer, 2019; Han, 2021a). The involvement and dedication of learners in school tasks and activities are known as behavioral engagement (Shappie and Debb, 2019).

### Resilience

Resilience refers to the defensive and weak components inside and outside an individual that influence the individual’s adaptation to alterations and traumatic encounters that result in an absence of homeostasis (Brewer et al., 2019). International students are encouraged to be resilient to manage and adjust to new environmental challenges. Resilient students are characterized by their capability of coping with an alteration. Therefore, resilience deals with how students bounce back or deal with difficult circumstances (Portnoy et al., 2018). Resilience is characterized as the inner ability to overcome hardships in unfavorable educational circumstances in the context of learning English (Shin and Kim, 2017). There are numerous ways in which scholastic resilience facilitates language learning. For instance, it improves learners’ inspiration to learn English (Martin and Marsh, 2006). Furthermore, Waxman et al. (2012) discovered that highly resilient and average resilient language students are more competitive in the class as opposed to non-resilient ones. Resilience is a developmental term that describes a person’s capability of recovering from misfortune and anxiety, assists with the administration of scholastic needs, advances scholastic success, and enhances the cycle of dealing with scholastic pressures (Brewer et al., 2019). Resilient learners are believed to perform better than non-resilient or mindless ones (Kim et al., 2019). In other words, resilience influences the quality of education and the overall improvement of individuals in various disciplines (Nolan et al., 2014). Resilience has three fundamental aspects, namely the capability of changing and adjusting as needed, the capability of being “elastic” and bouncing back swiftly from alterations, hardships, or restrictions, and the capability of staying confident and strong after changes (Schelvis et al., 2014; Xue, 2021).

### Tolerance of Ambiguity

Tolerance for ambiguity is an emotional element that is characterized as the capability of managing ambiguous new prompts without feeling frustrated and without making urgent requests to officials (Ehrman et al., 2003). Numerous scholars today have different characterizations for the notion of tolerance for ambiguity. As a mental concept, it is characterized as a person’s connection to an ambiguous incentive or occurrence. Ambiguity may be characterized as the unpredictability of language learning circumstances, normally triggered by the incapability of deciding the suitable context for clues or other incentives in a particular circumstance, where ambiguity is found in a new, intricate, or clashing circumstance (Nezhad et al., 2013; Han, 2021b).

In addition, many scholars have discovered that tolerance for ambiguity may be considered as one of the fundamental aspects used to define a person’s personality (Li and He, 2016). Tolerance for ambiguity means that a person encounters intricate new circumstances and embraces them without feeling frustrated. Ambiguous circumstances are ones about which a person does not have enough information. The capability of recognizing ambiguity in knowledge and practice impartially and openly is known as ambiguity tolerance. Those who tolerate ambiguity can take pleasure in imaginative possibilities without being intellectually or emotionally affected by ambiguity or unpredictability (Hadley, 2003). When a student encounters a high load of new information or clashes in language education, it can result in strong deconstructive emotional responses like stress. Tolerance for ambiguity generalizes to different dimensions of a person’s emotional and intellectual function and is distinguished by intellectual style, conviction and perspective frameworks, relational and social functions, and problem-solving practices (Furnham and Marks, 2013).

## Implications and Future Directions

The objective of the current study is to focus on the significance of enlightening, promoting, and nurturing learners’ engagement in education and teaching progressions. Learners’ engagement is viewed as one of the central and dominant aspects that should be taken into granted in the progress of an actual course, particularly with a focus on enhancing learners’ success and as it meaningfully helps learners’ academic success; it makes education more probable and it assists the prediction of learners’ educational performance and general development (Reeve, 2012). In summary, the results of this research emphasize the importance of promoting students’ engagement in the educational and teaching cycles. Since a successful learning framework needs to ensure growth during education and improve learners’ performance, tolerance for ambiguity and resilience must be practiced in the context of learning.

Unquestionably, the lack of tolerance of ambiguity and resilience in learners is felt in the educational systems. Considering the review of the related literature, some pedagogical implications can be recommended which might be regarded as significant. Teachers can relate the consequences of resilience and ambiguity tolerance-related inquires in their classrooms and train students that can be ready to be engaged in the classroom. Moreover, EFL educators carry a heavy load on their shoulders to create a more humanistic and less stress-inducing class setting that would assist their students with encountering less stress in the language class setting, thereby allowing them to be engaged in the class. Educators are encouraged to consider learners’ tolerance for ambiguity and resilience in education to develop a student-friendly educational setting that motivates their learners to be involved in the classroom. Recognizing the powerful impact of tolerance for ambiguity on foreign language education is very valuable and must result in educators changing how they arrange and carry out courses to assist students with overcoming mental impediments in a better way. Students will be more at ease, more confident, more encouraged, and more enthusiastic in the language class when they have enough information about what is happening in the class (Dornyei and Ryan, 2015).

In addition, the results can be very helpful to EFL students because having tolerance and resilience inspires and allows them to be more objective-centric and effective in education and/or acquiring L2. Learners who tolerate ambiguity are more flexible and able to deal with intellectual intricacy (Edison and Geissler, 2003). Furthermore, they are keen on using the latest intricate, clashing, and ambiguous technology and are more capable of controlling their educational cycle and making the right decisions. Therefore, their performance is anticipated to be better, which in turn enhances their achievement and perspective of this method of education. Individuals who are less tolerant of ambiguity feel nervous and keep away from ambiguous circumstances, while those who are more tolerant of ambiguity discern ambiguous circumstances as interesting and challenging (Hosseini Fatemi et al., 2016). Constructive emotions are more often seen in people with high resilience than in those with low resilience. Less resilient people respond more to daily incentives, making it harder to control deconstructive emotions. Resilient learners maintain a high level of inspiration for success and achievement despite anxiety-provoking occurrences and situations that result in poor school performance, and eventually, dropouts. Resilience assists individuals to deal with problems, and it promotes people’s capability to cope with challenges through determinations to infer hardship confidently instead of giving up (Kim et al., 2019).

Tolerance in language education could be interpreted as the capability of an individual to cope with new obscure incentives without frustration or anxiety. Therefore, having a high tolerance for ambiguity has many advantages that help learners have greater self-esteem in their social associations. Individuals who are highly tolerant of ambiguity can continue their discussion with confidence, even if there are words that are foreign and incomprehensible to them (Kurniasari and Indriani, 2021). For example, learners who are more tolerant of ambiguity may have learned to deal with a circumstance where they do not have a complete understanding of the comprehensible input they encounter. The low degree of ambiguity does not have a positive effect on the students since it makes them feel less assertive to transfer their thoughts and notions in the classroom. Ambiguity tolerance has an important part in the problem-solving and decision-making cycles, assisting students with performing better in intricate scenarios. Learners who tolerate ambiguity are more confident in their decisions, improve performance, and focus on advantageous results (Arquero et al., 2017). Based on the suppositions associated with the current review, it is advisable to encourage and advance the tolerance for ambiguity during foreign language learning to equip EFL students with the abilities and method styles that enable them to engage effectively in foreign languages.

English as a foreign language material developers are greatly suggested to incorporate content materials that motivate students and reinforce their resilience followed by the ambiguity of tolerance. The outcomes demonstrate that language learning decision-makers suggest more humane methods for language education to syllabus designers and educational organizations to develop an ambiance without anxiety in reading materials and to lessen the stress of EFL students. This would ultimately result in greater degrees of tolerance for ambiguity and resilience in the language education cycle that leads to their engagement, as well.

The present paper directed only English language university scholars. Nevertheless, the dearth of investigation in other areas, further education research is required to investigate the relationship among numerous issues helpful to learning at diverse stages of EFL education, and at private organizations, and even among English teachers. Future study is suggested to explore the relationship among the variables premeditated in this paper in accounting for language achievement both directly and indirectly. Moreover, more empirical research with a mixed-methods study comprising quantitative data related to the ambiguity of tolerance, resilience, and engagement, together with qualitative data collection techniques can be conducted to allow for studies that provide a prolonged perspective of the topic and lessen any dispositions.

## Ethics Statement

The studies involving human participants were reviewed and approved by the Baoding University Research Ethics Committee. The patients/participants provided their written informed consent to participate in this study.

## Author Contributions

MY, HW, and GX read the relevant literature and illuminated the role of ambiguity of tolerance and resilience on students’ engagement. All authors contributed to the article and approved the submitted version.

## Conflict of Interest

The authors declare that the research was conducted in the absence of any commercial or financial relationships that could be construed as a potential conflict of interest.

## Publisher’s Note

All claims expressed in this article are solely those of the authors and do not necessarily represent those of their affiliated organizations, or those of the publisher, the editors and the reviewers. Any product that may be evaluated in this article, or claim that may be made by its manufacturer, is not guaranteed or endorsed by the publisher.
